# Gamma Nail Combined with One Cannulated Compression Screw Fixation for Treating Pauwels Type III Femoral Neck Fractures in Young and Middle‐Aged Adults: Clinical Follow‐Up and Biomechanical Studies

**DOI:** 10.1111/os.13683

**Published:** 2023-02-27

**Authors:** Qiangqiang Wen, Feng Gu, Zilong Su, Ke Zhang, Xiaoping Xie, Jiangbi Li, Zhenjiang Sui, Tiecheng Yu

**Affiliations:** ^1^ Department of Orthopedics First Hospital of Jilin University changchun jilin China; ^2^ Department of Orthopedics, Xi'an No.3 Hospital the Affiliated Hospital of Northwest University Xi'an Shaanxi China

**Keywords:** Biomechanics, Cannulated Compression Screw, Femoral Neck Fracture, Internal Fixation, Intramedullary Nail

## Abstract

**Objective:**

Recently, some clinical studies have reported the use of an intramedullary nailing system for treating unstable femoral neck fractures or femoral neck fractures combined with femoral shaft fractures in young adults, and the results have indicated certain advantages. However, no study has investigated the mechanical properties of this method. We aimed to evaluate the mechanical stability and clinical efficacy of the Gamma nail combined with one cannulated compression screw (CCS) fixation for treating Pauwels type III femoral neck fracture in young and middle‐aged adults.

**Methods:**

This study consists of two parts: a clinical retrospective study and randomized controlled biomechanical test. Twelve adult cadaver femora were used to test and compare the biomechanical properties among three fixation methods: three parallel CCS (group A), Gamma nail (group B), and Gamma nail combined with one cannulated compression screw (group C). The single continuous compression test, cyclic load test, and ultimate vertical load test were used to evaluate the biomechanical performance of the three fixation methods. We also conducted a retrospective study of 31 patients with Pauwels type III femoral neck fractures, including 16 patients with fractures fixed with three parallel CCS (CCS group) and 15 patients with fractures fixed with Gamma nail combined with one CCS (Gamma nail + CCS group). The patients were followed up for at least 3 years, and all were evaluated for surgical time (from skin incision to closure), surgical blood loss, hospital stay, and the Harris hip score.

**Results:**

In mechanical experiments, we have found that the mechanical advantages of Gamma nail fixation are not as good as those of conventional CCS fixation. However, the mechanical properties of Gamma nail fixation combined with one cannulated screw perpendicular to the fracture line are much better than those of Gamma nail fixation and CCS fixation. No significant difference was found in the incidence of femoral head necrosis and nonunion between the CCS and Gamma nail + CCS groups. Moreover, there was no statistically significant difference in the Harris hip scores between the two groups. One patient in the CCS group showed significant withdrawal of cannulated screws at 5 months after surgery, whereas in the Gamma nail + CCS group, all patients, including those with femoral neck necrosis, showed no loss of stability of the fixation.

**Conclusion:**

Among the two fixation methods evaluated in this study, Gamma nail combined with one CCS fixation showed better biomechanical properties and may reduce complications associated with unstable fixation devices.

## Graphical abstract

Gamma nail combined with one cannulated compression screw fixation has the best biomechanical properties compared with three cannulated compression screw fixations for Pauwels type III femoral neck fracture, and may reduce complications associated with unstable fixation devices.

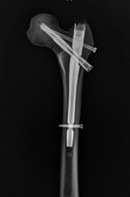

## Introduction

The incidence of femoral neck fracture, as an intracapsular fracture, has been increasing annually, particularly in young people.^1^, ^2^, ^3^ In young adults, most femoral neck fractures are vertically unstable shear fractures (Pauwels type III femoral neck fracture), with a high incidence of postoperative femoral head ischemia and necrosis.^4^ Anatomic reduction and stable internal fixation are among the principles for treating these fractures.^5^

Stable fixation of fractures requires a reliable internal fixation device. At present, the most common implants include multiple cannulated screws and dynamic hip screw (DHS).^6^, ^7^, ^8^ However, the incidence of complications, such as nonunion, varus collapse, shortening, and osteonecrosis, has been reported to be as high as 27%.^9^ A classic method for treating femoral neck fractures is internal fixation of three cannulated compression screws (CCS) placed in an inverted triangle pattern, which results in less trauma and exhibits good antitorsion ability.^10^ However, because CCS fixation cannot maintain the collodiaphyseal angle of the femur and cannot effectively resist shear force and prevent varus collapse and posterior varus, it is associated with a higher incidence of complications, such as nail withdrawal and fracture nonunion, after Pauwels type III fracture of the femoral neck.^11^, ^12^ Additionally, the lack of parallelism between multiple screws can adversely affect the compression force at the fracture site.^13^ Although DHS can maintain the collodiaphyseal angle of the femur and has a relatively low incidence of complications such as postoperative varus collapse and posterior varus, clinicians avoid using DHS because of its poor antirotation effect and increased probability of postoperative necrosis of the femoral head.^11^, ^12^ Complications associated with the existing fixation technique and unclear upper fixation methods indicate the need for further investigation of these fracture modes, surgical fixation techniques, and implants as well as the development of new methods and techniques to improve the treatment effect.^5^

A previous study showed that the choice of internal fixation device mainly depends on the type of fracture, specific location of the fracture, and Pauwels angle. The fracture pattern is the most important determinant of implant selection.^11^ Recently, some clinical studies have reported the use of an intramedullary nailing system for treating unstable femoral neck fractures or femoral neck fractures combined with femoral shaft fractures in young adults, and the results have indicated certain advantages.^14^, ^15^ The primary biomechanical advantages of intramedullary nails for treating femoral neck fractures include maintaining the collodiaphyseal angle and stabilizing the fracture while distributing the load along the femoral shaft, which are more physiologically consistent, thus enabling patients to get out of bed and move early in the postoperative period.^11^ Therefore, the Gamma nail, as a common clinically used intramedullary nail product, was used for treating femoral neck fractures in young and middle‐aged patients. However, no studies have compared the efficacy of Gamma nails with CCS fixation in treating femoral neck fractures. The objectives of our study were: (i) to study the mechanical properties of the Gamma nail fixation; (ii) to study the mechanical properties of the Gamma nail fixation + CCS fixation; (iii) to study the clinical effects of Gamma nail + CCS fixation in the treatment of Pauwels type III femoral neck fractures; (iv) technical application prospects of the Gamma nail + CCS fixation.

## Materials and Methods

### Ethics Statement

This study was performed in accordance with the guidelines adopted by the First Hospital of Jilin University for ensuring care of human subjects; the study protocol was approved by the Research Ethics Committee of the First Hospital of Jilin University (ref. no.2020–638). Written informed consent was obtained from all participants.

### Preparation of Specimens

Twelve male adult femoral cadaveric specimens (soaked in formalin for 4–9 months) from six patients aged 32–60 years (average age, 39 years) were selected. The soft tissue attached to the femur was excluded, and all femur specimens were devoid of fracture, arthritis, tumor, tuberculosis, rheumatism, and other pathological conditions, as confirmed via X‐ray photography. The 12 specimens were randomly categorized into groups A, B, or C, with four specimens in each group labeled together. The femoral neck diameters of each group were measured and compared, and the results revealed that the diameters of the three groups were 30.5 ± 1.6, 29.9 ± 1.3, and 29.85 ± 2.0 mm, respectively, with no statistically significant difference among the three groups (p > 0.05). The specimens were then wrapped in gauze soaked with 0.9% NaCl solution and stored in a freezer at −20°C. These were placed at room temperature for ablation 12 h before use. In the experiment, all femur specimens were sown off the distal end of the same part, and the processed femur specimens were embedded with denture powder to become fixed and form a base. The shaft of the femur was made at an angle of 15° to the natural vertical plane to simulate one‐foot bearing standing. A mark was made in the center of the femoral head of all specimens to observe and record the central displacement of the femoral head during the test.

### Fracture Model Preparation and Fixation

**Fig. 1:**
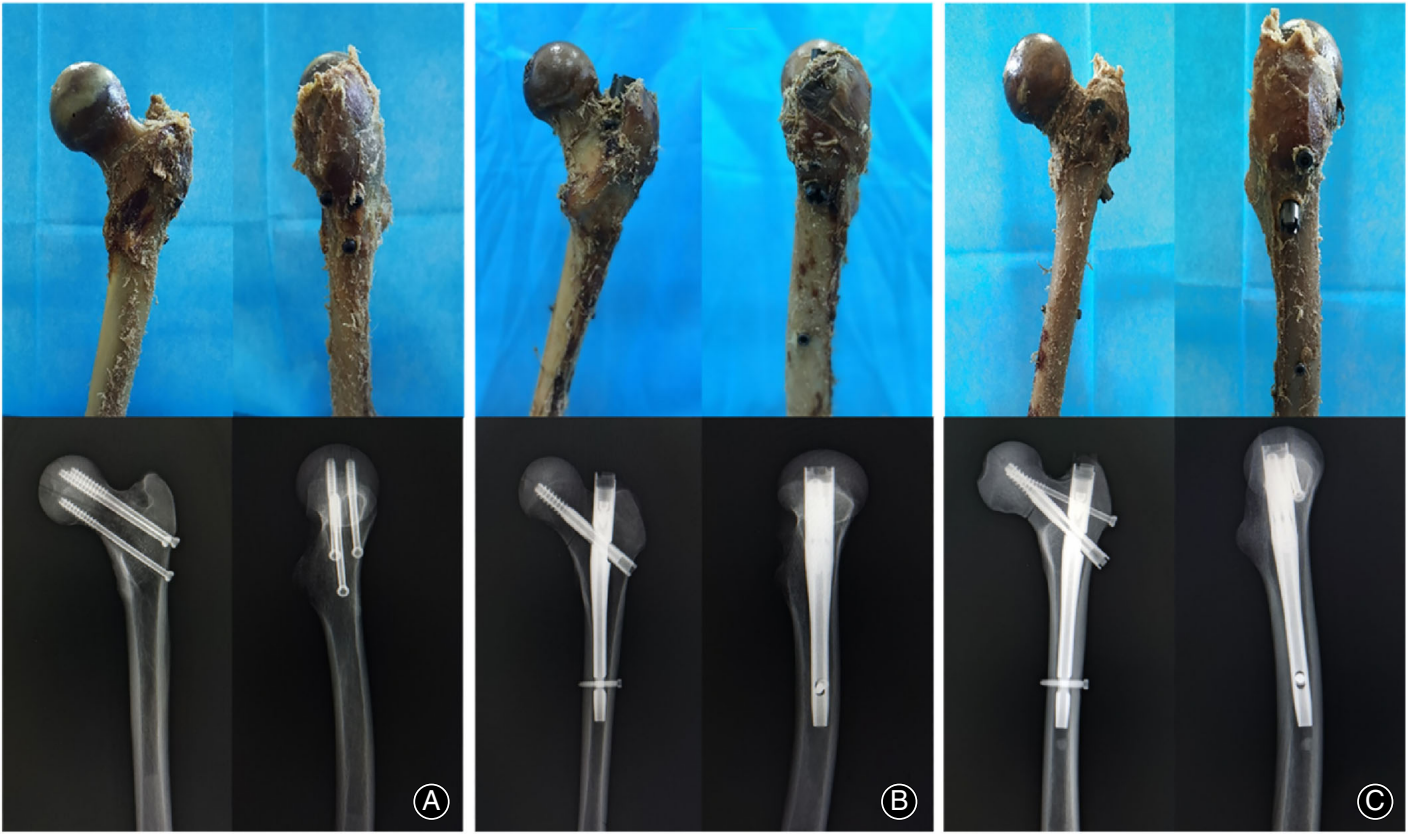
Photographs and radiographs of the three groups of internal fixation specimens. (A) CCS fixation group; (B) Gamma nail fixation group; (C) Gamma nail + CCS fixation group

To create Pauwels type III femoral neck fracture specimens, the femoral specimens were obtained and cut off with a hacksaw in the central part of the femoral neck at a shear angle of 70°. After anatomical reduction, group A specimens were fixed with three parallel CCSs placed in an inverted triangle pattern, group B specimens were fixed with Gamma nail, and group C specimens were fixed with Gamma nail combined with one CCS perpendicular to the fracture line. The same surgeon performed all operations. The three abovementioned internal fixation positions were verified using an X‐ray camera (Figure 1).

### Single Continuous Compression Test

We fixed the distal femur denture foundation base of the specimen on the base platform of CCS0‐44100 electronic universal testing machine. The joint surface of the femoral head was in contact with the custom concave hemispheric die to achieve uniform force. Before the formal test, a force of 200 N was preloaded three times to eliminate the effects of femur relaxation and creep, and the formal subsequent loading test was performed. Vertical compression loads (up to 1000 N) were applied to the femoral head at a speed of 0.1 mm/s. The displacement of the femoral head at 400, 600, 800, and 1000 N was recorded, and the axial stiffness of each group at 800 N was estimated. We calculated the axial stiffness of the femoral head (where axial stiffness refers to the ability of the femoral head to resist axial deformation under vertical load) using the following formula: axial stiffness = axial load/longitudinal displacement.

### Cyclic Load Test

The specimen used in the previous test was still firmly fixed on the universal testing machine. For the cyclic loading test, a cyclic vertical load was applied to the specimen at a speed of 0.6 mm/s, with a cyclic force of 100–1000 N and frequency of 0.2 Hz. There were 50 cycles in each group. The load was removed, and the displacement of the femoral head was recorded after completing the cyclic loading test.

### Ultimate Vertical Load Test

The specimen used in the previous test was firmly fixed on the universal testing machine. The specimen was subjected to the ultimate vertical load at a velocity of 0.6 mm/s until failure of internal fixation. The failure criteria were as follows: fracture of the femoral neck and head, sudden fluctuation of the load–displacement curve, and loss of internal fixation stability. The maximum load values for each group were recorded.

### Patients and Follow‐Up

We retrospectively reviewed young and middle‐aged patients with Pauwels type III femoral neck fracture who were treated with three CCSs or Gamma nail combined with one CCS at the First Hospital of Jilin University from 2015 to 2018.

The inclusion criteria were as follows: (1) age <65 years; (2) Pauwels type III femoral neck fracture; (3) complete follow‐up data over >3 years; and (4) Garden III/IV femoral neck fracture.

The exclusion criteria were as follows: (1) combined with ipsilateral intertrochanteric, subtrochanteric, or femoral shaft fractures; (2) long‐term use of high‐dose hormones or alcoholism; (3) pathological femoral neck fracture.

**Fig. 2:**
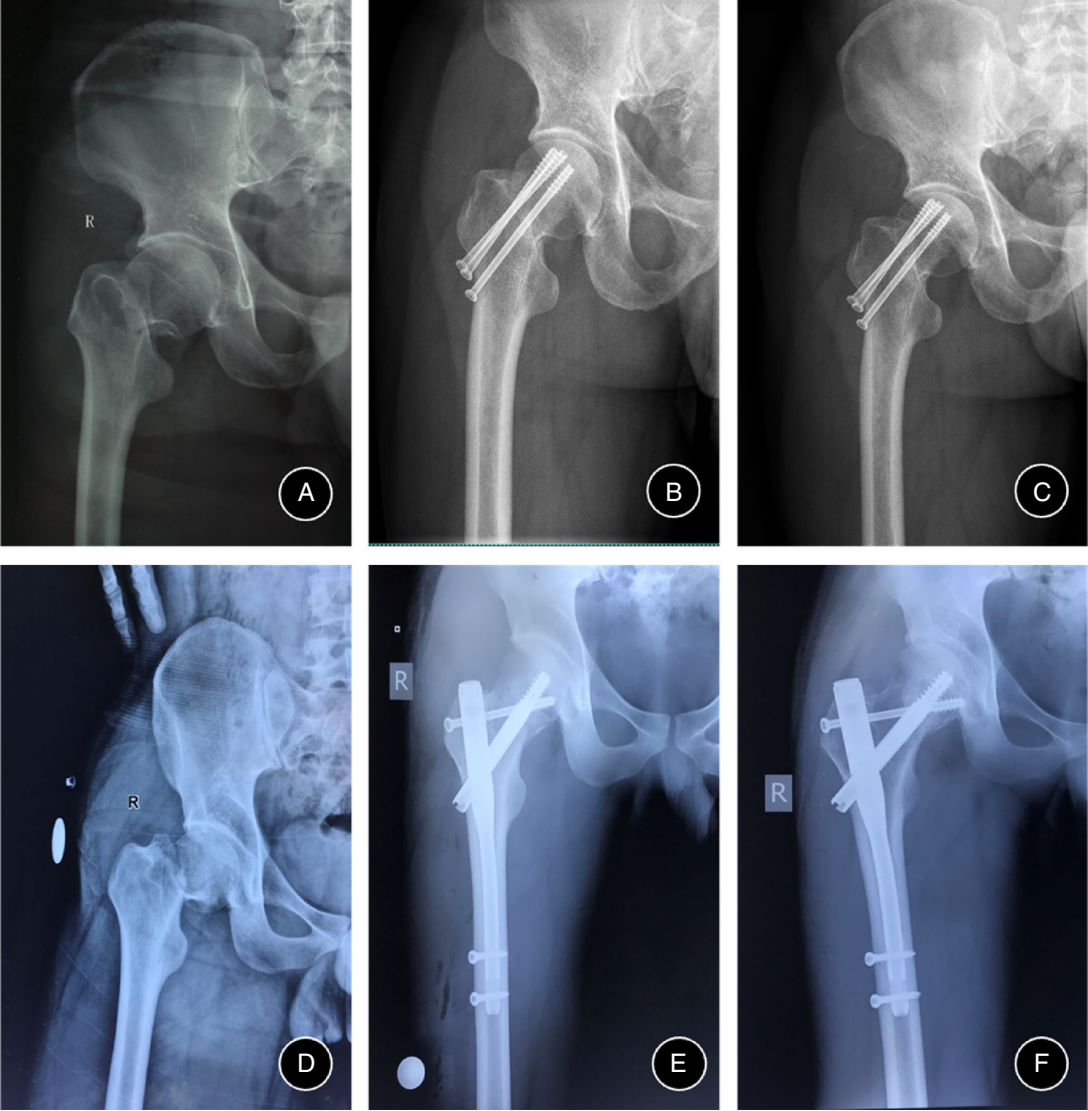
Typical radiographs of femoral neck fractures with success in the CCS group and the Gamma nail + CCS group. (A–C) Radiographs of injury, post‐operation, and 3 months after operation in the CCS group. (D–F) Radiographs of injury, post‐operation, and 3 months after operation in the Gamma nail + CCS group

Patients were classified into two groups: three CCS (CCS group) and Gamma nail combined with one CCS (Gamma nail + CCS group). In the CCS group, three CCSs were inserted in an inverted triangle pattern for treating the patients. The surgical fixation method used for treating patients in the Gamma nail + CCS group was as follows: first, the clinical routine implantation of the Gamma nail was performed, followed by the implantation of CCS behind the head screw of the Gamma nail (Figure 2). The quality of the reduction was assessed using Garden's alignment index combined with Lowell's curve. The angle between the medial trabecular stream in the femoral head and the medial cortex of the femoral shaft (trabecular‐shaft angle) was greater than 155° and smaller than 180° in both anteroposterior and lateral radiographs in both groups.^18^ The same surgeon performed all procedures. Passive range‐of‐motion exercises of the knee and hip were performed after the operation, with no weight bearing for 1.5 months. Patients were followed‐up clinically and radiologically every 2 or 3 months to assess healing progression and possible complications. The criteria for nonunion include pain at the fracture site, hypertrophy or atrophy of the fracture end as shown on radiographs, and the lack of spontaneous healing after 3 months of follow‐up.^16^ The evaluation of femoral head necrosis is mainly based on clinical symptoms and imaging for comprehensive diagnosis^17^ (especially magnetic resonance imaging and computed tomography). Because avascular necrosis of the femoral head usually occurs 2–3 years after operation, we followed the patient for 3 years. The Harris hip score was obtained 12 months after surgery. Data regarding surgical time (from skin incision to closure), surgical blood loss, and hospital stay were extracted from the medical records.

### Statistical Analysis

We used SPSS 18.0 (IBM Corp, Somers, NY, USA) software for statistical analysis. Measurement data were expressed as $\bar{x}$ ± s (mean ± standard deviation). The mechanical data were statistically compared using one‐way analysis of variance and pairwise LSD‐T test. We used independent sample *t*‐test for analyzing the clinical measurement data. Further, χ^2^ test was used to examine the differences between categorical variables, and Mann–Whitney *U* test was used to compare the Harris hip scores. A p‐value of <0.05 was considered to indicate statistical significance.

## Results

### Single Continuous Compression Test

**Fig. 3:**
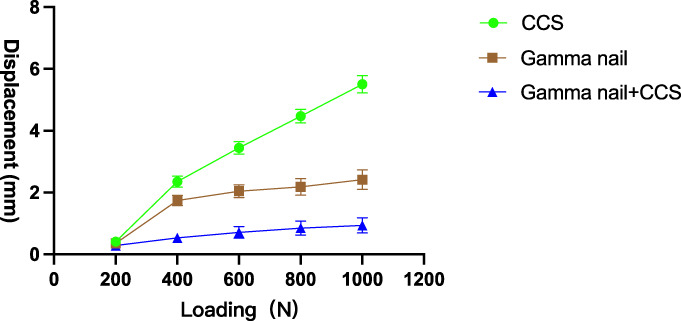
The load–displacement curves among the three groups

In the single continuous compression test, no specimen showed loss of stability. The increase in displacement was not linear with the load. The descending displacement of the femoral head showed variation with a load of 200–1000 N (Figure 3). When the load was 200 N, no significant difference was observed in the displacement of fixations between groups A and B (p = 0.50), A and C (p = 0.06), and B and C (p = 0.17). When the load ranged from 400 to 1000 N, the displacement of the fixations in group C was smaller than that in groups A and B in terms of single compressive resistance under various levels of loads (p < 0.001), and that in group B was lower than that in group A (p ≤ 0.001). Concurrently, we calculated the axial stiffness of each group at 800 N and revealed that the axial stiffness of group C (1006 ± 293 N/mm) was significantly larger than that of groups A (179 ± 9 N/mm; p < 0.001) and B (371 ± 47 N/mm; p = 0.001), with statistically significant differences.

### Cyclic Load Test

At the end of the cyclic load test, the load was removed with no loss of stability. Displacement data were recorded for each group. The femoral head displacement in group C (0.38 ± 0.15 mm) was significantly lower than that in groups A (2.56 ± 0.22 mm; p < 0.001) and B (2.35 ± 0.28 mm; p < 0.001), and the differences were statistically significant. However, no statistically significant difference was noted between groups A and B (p > 0.05).

### Ultimate Vertical Load Test

**Fig. 4:**
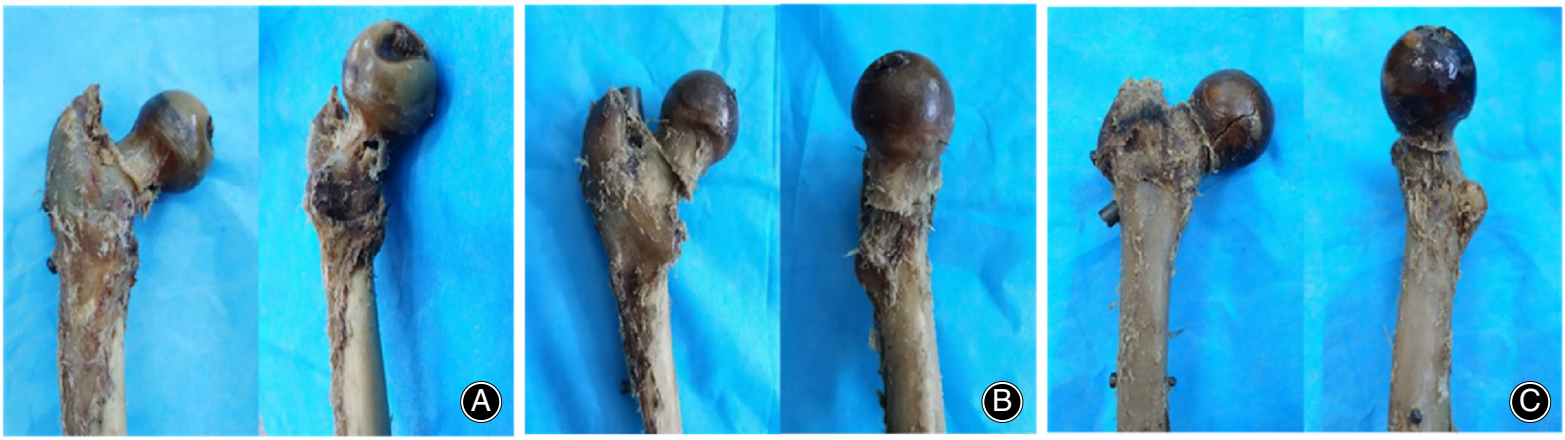
Specimens of the three groups after the ultimate vertical load test. (A) CCS fixation group; (B) Gamma nail fixation group; (C) Gamma nail + CCS fixation group

The ultimate vertical load test revealed that the load values of group C (3625 ± 167 N) were higher than those of groups A (2940 ± 276 N; p = 0.001) and B (2338 ± 17 N; p < 0.001), among which those of group A were higher than those of group B (p = 0.003). Figure 4 presents the final damage of the femoral head in each group. We observed a significant rotation of the femoral head in group B, whereas no obvious rotation of the femoral head was noted in groups A and C.

### Clinical Outcomes

Thirty‐six patients were included in the clinical research; however, five patients were lost to follow‐up. There was no significant difference in age, body mass index (BMI), gender, or Garden classification among the three groups (Table 1).

**Table 1:** General information and clinical outcomes during the perioperative period

	CCS (n = 16)	Gamma nail+ CCS (n = 15)	t/x^2^	p
Age (years)	50.56 ± 11.22	44.73 ± 14.16	1.28	0.21
BMI	23.20 ± 2.88	24.15 ± 3.38	−0.84	0.41
Male/female	10/6	8/7	0.27	0.60
Garden classification				
III	9	6	0.82	0.37
IV	7	9		
Operati on time(min)	73.94 ± 29.69	111.6 ± 29.62	−3.53	0.001*
Blood loss(ml)	65.0 ± 25.23	136.3 ± 27.02	−7.60	< 0.001*
Hospital stay(d)	5.13 ± 1.93	9.00 ± 3.85	−3.50	0.002*
Harris scores (*M* (*P*_*25*_ *, P*_*75*_))	83.00 (78.50, 90.75)	86.00 (83.00, 90.00)	‐	0.52
Complications				
Femoral head necrosis	2	1	0.30	0.58
Nonunion	0	0		

Abbreviations: CCS, three parallel cannulated compression screws Fixation; Gamma nail+ CCS, Gamma Nail Combined with One Cannulated Compression Screw Fixation; BMI, body mass index.

Table 1 shows the clinical outcomes of patients in the perioperative period. Compared with the Gamma nail group, the CCS fixation group showed advantages in operation time, intraoperative bleeding loss, and hospital stay (Table 1). There was no significant difference in the incidence of femoral head necrosis and nonunion between the two groups (Table 1). At 12 months postoperatively, no statistically significant difference was observed in the Harris hip scores between the two groups (Table 1).

## Discussion

Through mechanical experiments and clinical trials, we have found that the mechanical advantages of Gamma nail fixation are not as good as those of conventional CCS fixation. However, the mechanical properties of Gamma nail fixation combined with one cannulated screw perpendicular to the fracture line are much better than those of Gamma nail fixation and CCS fixation. Given this, we further investigated the clinical efficacy of Gamma nail fixation with a cannulated screw and found that it was not clinically superior to CCS fixation.

### Mechanical Properties of the Gamma Nail Fixation

In this study, we tested the mechanical properties of CCS fixation, Gamma nail fixation, and Gamma nail + CCS fixation in three trials. In the single continuous test, Gamma nail fixation was superior to CCS. However, in the cyclic load test, Gamma nail fixation was not superior to CCS fixation, and even worse than CCS fixation in the ultimate load test and showed rotation of the femoral head. This result indicates that the mechanical advantage of simple Gamma nail fixation is not as good as CCS fixation. It was prone to femoral head spin, failing internal fixation after a prolonged weight load.

### Mechanical Properties of the Gamma Nail + CCS Fixation

Given this, we added one CCS perpendicular to the fracture line to the Gamma nail fixation and tested its mechanical properties. The results showed that Gamma nail + CCS fixation was significantly stronger than the other two groups in all three tests, and there was no femoral head rotation after the ultimate load test. Therefore, we conclude that Gamma nail combined with one CCS perpendicular to the fracture line can enhance the strong fixation effect of the Gamma nail while compensating for the poor antirotation effect, thus demonstrating a far better mechanical effect than the three CCSs.

### Clinical Effects of the Gamma Nail + CCS Fixation

In this clinical retrospective study, the Gamma nail + CCS group had a longer operation time, greater intraoperative blood loss, and longer hospital stay than the CCS group, which might be associated with medullary reaming, opened medullary cavity, and blood sinus in the proximal femoral cancellous bone.^19^

In addition, we found no significant difference in Harris hip scores at 12 months between the Gamma nail + CCS and CCS groups (p = 0.923). During follow‐up, we noted one case of femoral head necrosis in the Gamma nail + CCS group (15 patients) and two cases of femoral head necrosis in the CCS group (16 patients), with no nonunion in either group. No statistical difference was found between the two groups in terms of the rate of femoral head necrosis. Among patients with femoral neck necrosis in the CCS group, one patient showed significant withdrawal of the cannulated screws 5 months after surgery, whereas in the Gamma nail + CCS group, all patients, including those with femoral neck necrosis, showed no loss of stability of the fixation, indicating that a major advantage of Gamma nail combined with one CCS fixation was reducing the risk of femoral neck necrosis due to loss of stability of the internal fixation. The results of the retrospective follow‐up revealed that although Gamma nail combined with one CCS fixation caused more trauma than three CCS fixations, and was not inferior in terms of complications and function, it may reduce the complications caused by unstable instrumentation. Further, the cost of the equipment used for the Gamma nail combined with one CCS fixation is three to four times that of the three CCS fixations; thus, Gamma nail combined with one CCS fixation is only recommended for treating serious Pauwels type III femoral neck fractures.

### Technical Application Prospects of the Gamma Nail + CCS Fixation

The stability of femoral neck fixation after fracture is closely associated with postoperative complications, such as femoral head necrosis and recovery of joint function.^20^ In the Gamma nail system, the main nail is located in the medullary cavity, and the downward conduction force arm is closer to the femoral calcar. The asymmetrical groove design allows the lag screw to slide only outward and not medially; thus, it has superior mechanical stability.^14^, ^15^ However, our mechanical study found that the anti‐rotation effect of Gamma nail fixation was poor, which greatly weakened its mechanical properties. Gamma nail combined with a cannulated screw overcomes this disadvantage and exhibits greater mechanical properties than conventional CCS fixation. Our clinical study found no statistically significant difference between Gamma nail + CCS and CCS fixation in osteonecrosis of the femoral head, nonunion, and hip function scores. In contrast, Gamma nail + CCS was associated with longer operation time, longer hospital stay, and more intraoperative blood loss. We analyzed that this result occurred because the mechanical instability of the included femoral neck fracture model in our clinical trial did not fall within the dominant advantage interval between Gamma nail + CCS fixation and CCS fixation. Further, the cost of the equipment used for the Gamma nail combined with one CCS fixation is three to four times that of the three CCS fixation. Thus, Gamma nail combined with one CCS fixation is only recommended for treating serious Pauwels type III or femoral neck fractures combined with ipsilateral femoral shaft fractures.

### Strengths and Limitations

This is the first study to compare the mechanical properties and clinical results of Gamma nail combined with one cannulated compression screw in the treatment of femoral neck fractures, which provides a reference for the future use of Gamma nail in the treatment of severe femoral neck fractures.

The number of patients included in this study was small, and the long‐term effect of this fixation requires further research. In addition, other internal fixation devices such as the femoral neck system (FNS), four CCS, and DHS are also used in the treatment of unstable femoral neck fractures. Due to limited funds, these devices were not included in the trial control group for comparison, which requires further research in the future.

### Conclusions

According to the results of the biomechanical experiments and clinical studies, Gamma nail combined with one CCS fixation exhibits superior biomechanical characteristics compared with the traditional three CCS fixation, whereas single Gamma nail fixation has poor biomechanical stability and is difficult to use for treating Pauwels type III femoral neck fractures. Concurrently, Gamma nail combined with one CCS fixation is comparable with the traditional three CCS fixation in terms of functional recovery and complications and may even reduce complications caused by unstable internal fixation equipment. However, this method is associated with greater intraoperative bleeding and warrants a prolonged hospital stay; hence, this technology should be cautiously used in older patients.

#### Author's Contribution

Tiecheng Yu contributed to the conception of the study; Qiangqiang Wen performed the experiment; Feng Gu contributed significantly to analysis and manuscript preparation; Qiangqiang Wen performed the data analyses and wrote the manuscript; Zilong Su, Ke Zhang, Xiaoping Xie, Jiangbi Li and Zhenjiang Sui helped perform the analysis with constructive discussions. Qiangqiang Wen and Feng Gu contributed equally to the article.

## Conflicts of interest

There are no financial conflicts of interest to disclose for all authors in this article.

## Funding Information

This study was supported by the National Natural Science Foundation of China (Tiecheng Yu, Grant No. 31970090).

## IRB Information

This study was carried out in accordance with the guidelines adopted by the First Hospital of Jilin University; for ensuring care of human subjects. The study protocol was approved by the Research Ethics Committee of the First Hospital of Jilin University (ref. no. 2020–638). Written informed consent was obtained from all participants. The study was performed following the principles of the Declaration of Helsinki (as revised in Brazil in 2013).
